# The impact of the neisserial DNA uptake sequences on genome evolution and stability

**DOI:** 10.1186/gb-2008-9-3-r60

**Published:** 2008-03-26

**Authors:** Todd J Treangen, Ole Herman Ambur, Tone Tonjum, Eduardo PC Rocha

**Affiliations:** 1 Algorithms and Genetics Group, Department of Computer Science, Technical University of Catalonia, Jordi Girona Salgado, 1-3, E-08034 Barcelona, Spain; 2 Centre for Molecular Biology and Neuroscience and Institute of Microbiology, University of Oslo, Rikshospitalet, NO-0027 Oslo, Norway; 3 Centre for Molecular Biology and Neuroscience and Institute of Microbiology, Rikshospitalet Medical Centre, NO-0027 Oslo, Norway; 4 Atelier de Bioinformatique, UPMC - University of Paris 06, 4, Pl Jussieu, 75005 Paris, France; 5 Microbial Evolutionary Genomics Group, URA CNRS 2171, Institut Pasteur, 28 R. Dr Roux, 75015 Paris, France

## Abstract

**Background:**

Efficient natural transformation in *Neisseria *requires the presence of short DNA uptake sequences (DUSs). Doubts remain whether DUSs propagate by pure selfish molecular drive or are selected for 'safe sex' among conspecifics.

**Results:**

Six neisserial genomes were aligned to identify gene conversion fragments, DUS distribution, spacing, and conservation. We found a strong link between recombination and DUS: DUS spacing matches the size of conversion fragments; genomes with shorter conversion fragments have more DUSs and more conserved DUSs; and conversion fragments are enriched in DUSs. Many recent and singly occurring DUSs exhibit too high divergence with homologous sequences in other genomes to have arisen by point mutation, suggesting their appearance by recombination. DUSs are over-represented in the core genome, under-represented in regions under diversification, and absent in both recently acquired genes and recently lost core genes. This suggests that DUSs are implicated in genome stability rather than in generating adaptive variation. DUS elements are most frequent in the permissive locations of the core genome but are themselves highly conserved, undergoing mutation selection balance and/or molecular drive. Similar preliminary results were found for the functionally analogous uptake signal sequence in Pasteurellaceae.

**Conclusion:**

As do many other pathogens, *Neisseria *and Pasteurellaceae have hyperdynamic genomes that generate deleterious mutations by intrachromosomal recombination and by transient hypermutation. The results presented here suggest that transformation in *Neisseria *and Pasteurellaceae allows them to counteract the deleterious effects of genome instability in the core genome. Thus, rather than promoting hypervariation, bacterial sex could be regenerative.

## Abstract 2

A study of the origin and distribution of the abundant short DNA uptake sequence (DUS) in six genomes of Neisseria suggests that transformation and recombination are tightly linked in evolution and that recombination has a key role in the establishment of DUS.

## Background

The act of combining genetic information from two different individuals is ubiquitous among living organisms. Genetic exchange can take the form of sexual reproduction in some eukaryotes, whereas in most prokaryotes it is the result of horizontal transfer of DNA from a donor to a recipient cell. Horizontal transfer may result in the introduction of new and radically different genetic information or in the allelic replacement of existing genetic loci by homologous recombination. Among the three mechanisms that facilitate horizontal gene transfer (HGT), natural transformation is often referred to as the bacterial equivalent of meiotic sex in eukaryotes. This is because it re-assorts genetic information among members of the same species and, contrary to transduction and conjugation, is a process under the direct control of the recipient cell [1]. Because maintenance of the capacity for transformation is strictly dependent on its having positive effects on the fitness of the recipient bacteria, it might be regarded as the mechanism of choice for elucidating the advantages of sex in prokaryotes.

Many bacterial species are naturally competent for transformation, some constitutively so, whereas others are competent in response to specific environmental conditions [2]. Naturally competent bacteria have evolved mechanisms or strategies to avoid entry of heterologous and potentially harmful DNA [3]. Similar to reproductive barriers that exist between eukaryotic species, a preference for homologous DNA over heterologous DNA is evident in a range of competent species through adaptations including induction of competence by quorum sensing, presence of restriction modification systems, and blockage of heterologous recombination by stringent RecA function or mismatch repair [4].

Transformation in *Neisseria *spp. and members of the family Pasteurellaceae requires the presence of a specific DNA uptake sequence (DUS) [5] or uptake signal sequence (USS) [6,7], respectively, in the incoming DNA. These signals allow discrimination between DNA from closely related strains or species and foreign/unrelated DNA. The DUS of *Neisseria *spp. is a short signal extending 10 nucleotides: 5'-GCCGTCTGAA-3' [8]. It is present in approximately 2,000 copies occupying 1% of the sequenced neisserial genomes, which is much more than expected given the sizes of the genomes and their composition, and can only be maintained by strong counteraction to drift [9,10]. The efficacy of transformation is higher if the 10-mer DUS is preceded by an A and a T [11]. The 10-nucleotide signal is required and sufficient for transformation [11] and is the one considered in this study. However, because 75% of 10-nucleotide DUSs also are also extended 12-nucleotide DUSs, this should not affect our conclusions. DUSs often appear as closely spaced inverted repeats that function as rho-independent transcription terminators [11-13]. This local arrangement of inverted repeats does not lead to a change in the efficacy of transformation, which only depends on the presence of a single DUS [11].

Transformation has traditionally been studied and conceptualized as a succession of distinct stages: surface binding/entry through an outer membrane pore, transit across the periplasm and the inner membrane, and genome integration. However, recent studies have demonstrated that these processes, at least in *Bacillus subtilis*, are tightly linked in both space and time [14] and the term 'transformation complex' has been coined. DNA has, per definition, been taken up when it is no longer degradable by DNase, but more research is needed to appreciate fully the physical implications of the DNase protected state and exactly where DUS specificity acts.

Two major theories have been proposed to account for the origin and maintenance of DUS signals. Classically, DUSs have been regarded as cellular guardians that prevent the entry of potentially damaging non-DUS containing sequences, such as naked DNA from phages, plasmids, or transposable elements. Indeed, DUS specificity effectively disfavors DNA originating from distantly related species because these lack DUSs. It has also been suggested that DUS-specific transformation may lead to molecular drive [9]. If the DNA uptake machinery by some physical means has a preference for DUSs, then sequences containing a DUS are more likely to be transferred and, consequently, effectively accumulate in the genomes of *Neisseria*. At the extreme end of this concept, it has been suggested that DUSs increase in frequency purely because of molecular drive, independently of any putative positive effect on fitness (selfish DUS hypothesis) [15,16].

Molecular drive is a model of evolution that provides an explanation alternative to natural selection and is based on purely stochastic preferential uptake of DUS-containing DNA. Preferential DNA uptake is a biologic mechanism and should not be confused with molecular drive, which is a model of evolution and might be one of its consequences. The mechanism of preferential DNA uptake may also be involved in DUS/USS fixation by classic natural selection driven by the advantage of taking up conspecific DUS-containing DNA or preventing the entry of alien sequences. The darwinian model generally seeks the potential selective advantages of sex and particularly 'safe sex', whereas the molecular drive model seeks to explain how these genomes can tolerate such large amounts of an 'intrusive' repetitive sequence without discretion, and in essence how DUSs/USSs can accumulate without being positively selected for by forces affecting the fitness of the organism.

Competent bacteria have invested extensively in complex machineries to facilitate transformation, involving a comprehensive range of competence and recombination proteins [17]. *Neisseria *spp. express type IV pili that are required for transformation [18]. Furthermore, a type IV secretion system that exports DNA into the environment has been described in most gonococci and some strains of meningococci [19,20]. Thus, neisserial sex is an active process mediated by specific machineries that can import and export genetic information. Competence for transformation in *Neisseria *is constitutive throughout its growth cycle and does not depend on environmental conditions [21]. Studies of population structures, which in nature may range from complete clonality to panmixia, have shown that transformation in the pathogenic *Neisseria *has fuelled high rates of recombination [22]. It has been estimated that an allele of the *Neisseria meningitidis *genome is ten times more likely to change by recombination than by point mutation [23].

The reasons for such an intense recombination rate have often been associated with the lifestyle of *Neisseria *spp. and in particular with virulence in humans. Members of the genus *Neisseria *and the family Pasteurellaceae populate the mucosal surfaces of humans and animals. Of particular clinical significance are *N. meningitidis *and *Haemophilus influenzae*, which are leading causes of bacterial meningitis and septicemia worldwide [24], and *Neisseria gonorrhoeae*, which causes the sexually transmitted disease gonorrhea [25]. The commensal *Neisseria lactamica *is commonly found in the upper respiratory tract of young children and teenagers and may contribute to immunity to meningococcal disease [26]. Analyses of the four published neisserial genomes revealed high densities of repeated elements [27-30]. Intrachromosomal recombination between these repeats is a major source of variability in *Neisseria*, resulting in frequent adaptive changes in gene expression profiles [31,32] and even re-occurring states of hypermutability [33,34].

Given the role of HGT in genome fluidity, elucidation of the evolutionary role of natural transformation is pivotal to our understanding of prokaryotic adaptation. The abundant DUS and USS elements are required for efficient natural transformation in *Neisseria *and Pasteurellaceae members, respectively. If these repeat sequences are markers of selection for transformation, as commonly believed, then their differential presence and conservation across a genome may also contribute to our understanding of the advantages of sex, which is a longstanding question in evolutionary biology [35,36]. Here, we used the potential provided by the availability of six complete neisserial genomes to align globally the core genome and to define the sets of genes that are ubiquitous and those that were recently acquired or recently lost in each group. These multiple genome alignments warrant a new and powerful approach to address the puzzle of the origin and fate of DUSs in these genomes and the association between these signals and recombination events. In this work we use the term 'recombination' for the process of homologous recombination between the chromosome and DNA from other cells. A striking correlation between the average distance between DUSs and the length of conversion fragments was found, which indicates that the process of transformation is tightly linked to and even shaped by recombination. The presence of unique DUSs that interrupt otherwise conserved regions in neisserial alignments further emphasizes the role of recombination in DUS evolution. Within the limits of available data, we find similar results when analyzing the genome of *H. influenzae*. The findings presented here enhance the influence of allelic replacement as the bacterial equivalent of sex and the role of transformation in genome maintenance.

## Results

### Global genome alignments

**Figure 1:**
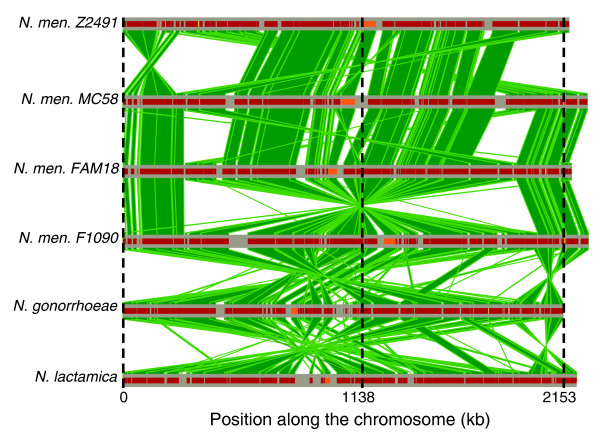
Visual representation of M-GCAT's multiple alignment of six *Neisseria *genomes. The horizontal lines correspond to a linear representation of each genome sequence. The vertical polygons represent the 79 M-GCAT clusters of average length 28,153 nucleotides. Evident from the visual representation of the alignment is that there are many rearrangements throughout the genome comparison. Inverted vertical polygons depict an inversion between two of the genome sequences. The small rectangles overlapping the horizontal lines correspond to the standardized MUSCLE alignment score for each respective M-GCAT cluster; darker intensities indicate better scores. In total, 82.5% of the original genome sequences are covered by the multiple alignment, and 17.5% was left unaligned. kb, kilobases.

We conducted two types of multiple alignments of the genomes of *N. meningitidis *Z2491 (serogroup A), MC58 (serogroup B), FAM18 and 8013 (serogroup C), *N. gonorrhoeae *FA1090, and *N. lactamica *ST-640 (see Materials and methods, below). First, the genes with orthologs in all of the six neisserial genomes were identified, translated, aligned with MUSCLE [37], and then back-translated to the original DNA sequence. These alignments are highly accurate at these phylogenetic distances [38] and were used to fine tune the parameters of the multiple genome comparison and alignment tool M-GCAT [39]. Second, we constructed a global multiple alignment of the six genomes (Figure 1) using M-GCAT. All but one M-GCAT cluster (collinear aligned region) yielded a high alignment score. Removing this region from the multiple alignments did not change the results. The sequences were highly similar, with an average protein similarity between orthologs of *N. meningitidis *and *N. gonorrhoeae *of about 97%, between *N. meningitidis *and *N. lactamica *of about 94%, and between *N. meningitidis *strains of more than 98.7%.

Despite this phylogenetic proximity, several rearrangements have accumulated after the divergence of these genomes [30]. This is a consequence of the high numbers of repeats that these genomes contain and requires the use of a multiple alignment method that handles rearrangements and duplications, such as M-GCAT. The global multiple alignment was composed of 79 co-linear regions, with breakpoints induced by the chromosomal rearrangements, and covered approximately 82.5% of each genome (Additional data file 1). Within these common regions there was a high percentage of monomorphic sites, and, overall, the multiple alignment contained homologous regions with high sequence identity present in all of the neisserial genomes. We also conducted a global alignment of four *H. influenzae *genomes that resulted in 53 co-linear alignment regions (Additional data file 6). The genomes of the other members of the Pasteurellaceae could not be globally aligned because of the relatively large phylogenetic distances involved. Because the multiple genome alignment in this clade only includes four closely related strains of a single species, the range of analysis that could be made was much more limited.

### The distribution of neisserial DUS corresponds to the length of conversion fragments

Because DUSs are required for transformation, a nonrandom positioning or conservation of this repeat (also USS) could pinpoint distinct effects on recombination and thus shed some light on the role(s) of bacterial sex. Within certain limits, a high local density of DUS is expected to increase the probability of transfer of the corresponding chromosomal region. A linear relationship between the affinity for DNA in transformation and the frequency of DUSs on the segment has indeed been demonstrated in a competitive assay [8]. However, only a single DUS is required for efficient transformation, and two very closely spaced motifs do not increase the transformation efficiency [11]. The usual interpretation of these results is that one DUS is enough for transformation, but because DNA is sheared in the environment a higher density of DUS increases the probability that a given fragment will contain a DUS and thus enter the cell and recombine. The positive effect of DUS density in conversion will become smaller with the increase in DUS density up to the point at which the selective effect is too weak to counterbalance drift. Selection for high DUS density will also depend on the size of DNA fragments that are taken in by the cell. If fragments are smaller for a species, then there should be compensatory selection for higher DUS density. One would thus expect that the limits of selection or molecular drive to increase DUS density were indicated by the distribution of sizes of conversion fragments. If conversion fragments are large, then a high DUS density would not be maintained. If, on the other hand, these fragments are small, then DUSs could be more tightly packed in the chromosome. If selection or molecular drive varies along the chromosome, then DUSs should not be homogeneously distributed throughout the genome, and more recombining regions should contain more of these elements.

**Figure 2:**
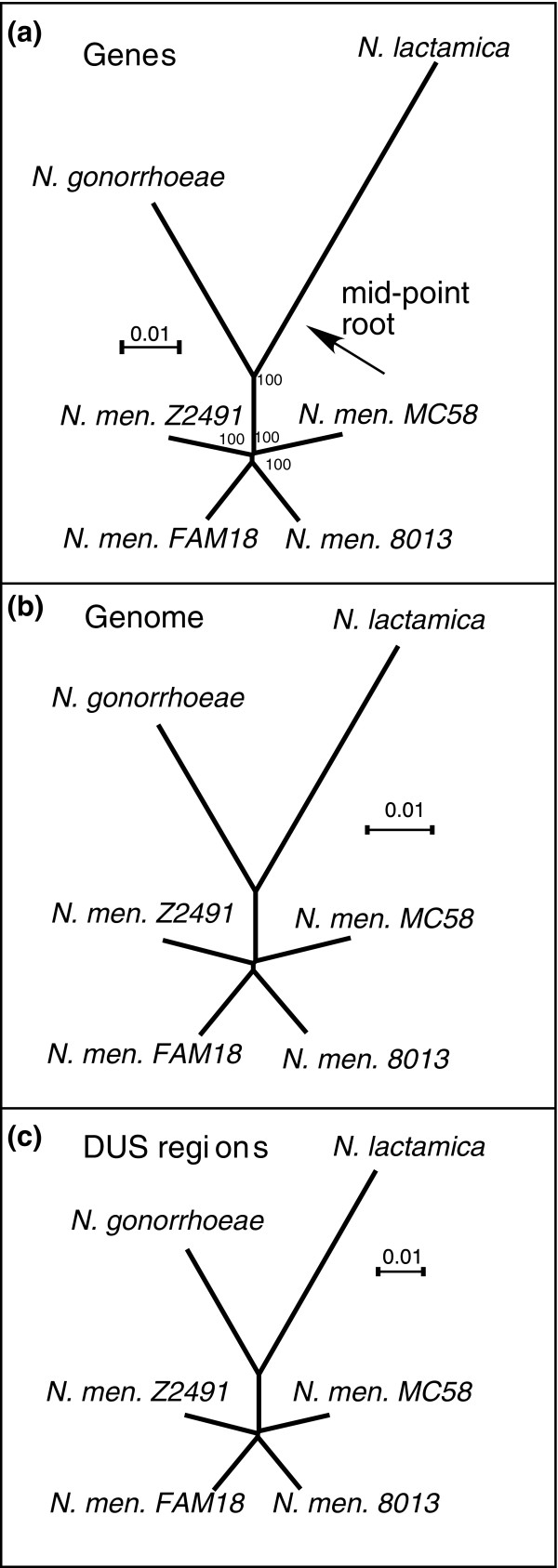
Consistent neisserial phylogenetic trees. **(a) **From the concatenated ubiquitous gene alignment (numbers indicate nonparametric bootstrap results in percentage out of 1,000 experiments); **(b) **from the concatenated 1,000 nucleotides regions (± 500 nucleotides) surrounding ubiquitous DNA uptake sequence (DUS) sites in the M-GCAT multiple alignment; and **(c) **entire concatenated M-GCAT multiple alignment. Distance matrices were computed from the alignments using Tree-Puzzle [85] by maximum likelihood with the HKY+Γ model and trees computed with BIONJ [86].

The genome sequencing projects for *N. meningitidis *were designed to embody the highest possible diversity in the species; specifically, the strains were chosen to be at the largest possible phylogenetic distance. This means that the phylogenetic tree of these genomes is expected to contain very short internal branches. This inference challenge is aggravated by the frequent recombination in the species, which also results in small internal branches [40]. Therefore, before assessing conversion fragments, we checked whether one could identify a robust phylogeny of the core genes of *Neisseria*. Phylogenetic trees of the clade were independently constructed using three partly overlapping datasets: the concatenate of all ubiquitous genes, the M-GCAT multiple genome alignment, and the concatenate of aligned DUS-containing regions (see Materials and methods, below). In all cases, highly robust and topologically identical trees were obtained, showing that - despite frequent recombination in *Neisseria *- one can identify a consensus phylogenetic tree that can be used as a reference guide for the detection of recombination events (Figure 2). The trees also showed that the average history, as traced by the ubiquitous genes, the multiple genome alignment, and the DUS-containing regions, is the same.

**Figure 3:**
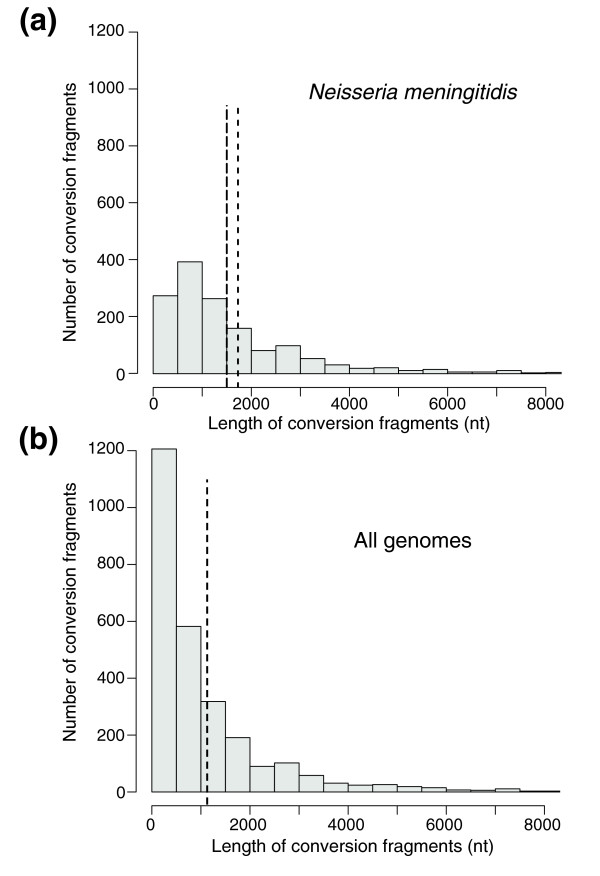
Distribution of lengths of gene conversion events. We used GENECONV to predict gene conversion events in each pair of sequences of the multiple alignment. **(a) **Gene conversion events were predicted only for *Neisseria meningitidis *sequence pairs (six total), in which we identified 1,469 gene conversion fragments with an average length of 1,728 nucleotides. **(b) **We identified 2,717 putative gene conversion fragments in all pairs of sequences (15 total) with an average length of 1,127 nucleotides (nt; indicated by the vertical dashed line). In both figures, the height of each bar in the figure indicates the number of gene conversion fragments with the specified length.

The M-GCAT multiple genome alignment was then searched for gene conversion events using GENECONV [41]. GENECONV is a computer program that applies statistical tests to identify the most likely candidates for gene conversion events between sequences in an alignment (see Materials and methods, below). The analysis was first restricted to the meningococcal genomes because this clade is more sampled. In this set, the average gene conversion fragment was 1,728 nucleotides long (Figure 3). Accounting for recombination in all of the neisserial genomes, and not only in *N. meningitidis*, reduced the size of the average conversion fragment to 1,127 nucleotides. The smaller size of the conversion fragments when including the more distantly related genomes may reflect the unavailability of longer segments of strict homology for homologous recombination or uptake of smaller DNA segments by the outgroups. Lower similarity might also bias GENECONV to detect smaller fragments preferentially. Published analysis of multilocus sequence typing data has yielded comparable results, namely that average neisserial conversion fragments vary between 500 and 2,500 nucleotides in length [42]. This may be an underestimation of the size of fragments when the density of conversion events is so high that events between different pairs of genomes overlap.

If DUSs are associated with recombination, either by selection or molecular drive, then one would expect there to be a close association between their spacing and the sizes of conversion fragments. We then computed the distribution of the distances between consecutive DUSs. To control for the selection of close inverted DUSs in transcription terminators, we clustered pairs of DUS in each such element into a composite DUS (cDUS). Regions containing a complementary DUS pair separated by 21 nucleotides or less were regarded as a single cDUS. Thus, the term cDUS stands for a single isolated DUS or a pair of DUSs in close inverse configuration in a rho-independent terminator. The average inter-cDUS distance among meningococci is approximately 1,500 nucleotides, which corresponds to the average size of conversion fragments (Additional data files 2 and 5). It should be noted that not controlling for pairs of DUSs in transcription terminators did not radically change this number (average distance between DUS about 1,150 nucleotides), emphasizing the close concordance between conversion fragments and DUS distribution. When all neisserial genomes were analyzed, the distance between cDUS decreased to 1,128 nucleotides because DUS density is higher in *N. lactamica *(Table 1). This higher density of DUSs in *N. lactamica *is in remarkable agreement with the conversion fragment data, which shows much shorter conversion fragments in this species than in *N. meningitidis *Z2491 (573 versus 1,734 nucleotides; *P *< 0.001, by Wilcoxon test). This suggests that shorter conversion fragments might lead to selection for higher density of DUSs. Taken together, all these results show a remarkable similarity between the average distance between cDUS (1,500 and 1,128 nucleotides when accounting for meningococcal or all genomes, respectively) and the sizes of conversion fragments (1,728 and 1,127 nucleotides, respectively), highlighting the close association between the two.

**Table 1:** Genome size, number of genes, and DUS 10-mers distribution in the genomes of *Neisseria*

Genome	Genome size (kb)	Number of genes	DUS distribution				
			
			% in genes	% alignment	CDUS	Total	DUS-1
*N. meningitis *Z2491	2,184	2,065	34.5	89.6	405	1,892	815
*N. meningitis *MC58	2,272	2,079	34.6	87.7	431	1,935	809
*N. meningitis *FAM18	2,194	1,976	34.0	89.8	431	1,888	818
*N. meningitis *8013	2,277	2,126	34.7	88.7	422	1,915	816
*N. gonorrhoeae*	2,153	2,185	38.6	89.9	396	1,965	774
*N. lactamica*	2,233	2,067	42.0	88.9	500	2,245	562

The table indicates the percentage of DNA uptake sequence (DUS) in genes, the percentage of DUSs in the M-GCAT multiple genome alignment, the number of composite DUSs (cDUS), the total number of DUSs in the genome, and the number of motifs matching the DUS consensus except at one position (DUS-1).

### The stringent conservation of DUS

The global multiple genome alignment allows the identification of DUSs located in regions that can be aligned, namely in the core genome, and to study how they changed over time. For this purpose, it suffices to identify the location of DUSs in the alignment and analyze the corresponding sequence columns. Previous studies have shown that some very distant orthologous genes maintain the presence of USSs in their sequences, but without precisely studying the conservation of the motif sequences [15]. In this study, the M-GCAT global genome alignment allowed precise prediction of the evolution of these elements in each of the aligned positions. DUSs were found to be highly conserved; in fact, they were much more conserved than the average conserved sequence (about 85% identity), exhibiting on average 97% sequence identity. Additionally, 71% of the DUSs in the multiple alignments were exactly conserved in all genomes. In the four *N. meningitidis *genomes, which are most closely related, the number of exactly conserved DUSs was greater than 90%.

**Figure 4:**
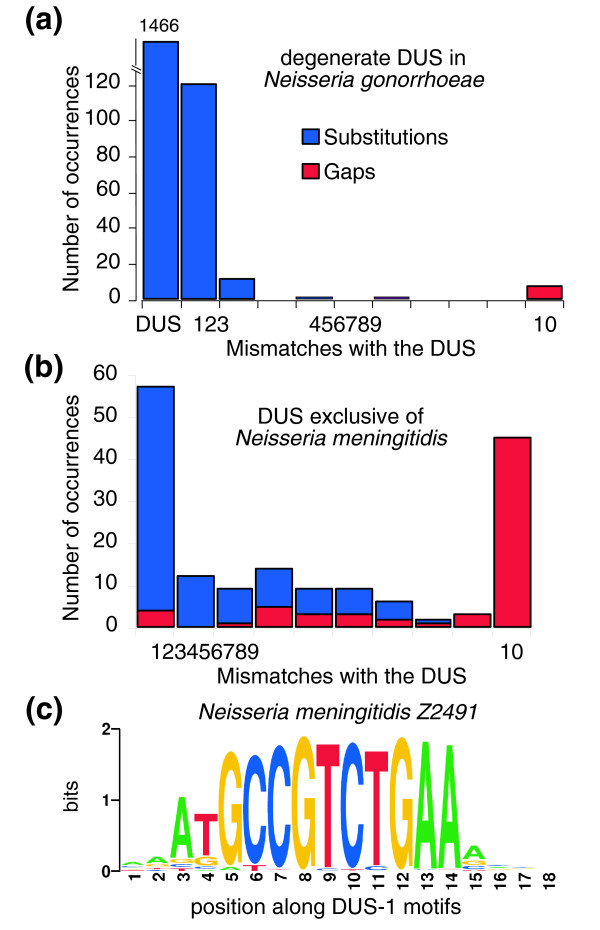
DUS degeneracy. **(a) **Histogram of the degeneracy of DNA uptake sequence (DUS) elements in *Neisseria gonorrhoeae *that are exact DUSs (nondegenerate) in all of the other five genomes. These DUSs have most likely degenerated in the *N. gonorrhoeae *lineage. The number of substitutions (blue striped bars) and gaps (red striped bars) present in each degenerate DUS site in *N. gonorrhoeae *were calculated. The x-axis labels are the number of each type of mutation for each case, for example 1 to 10 substitutions or gaps. **(b) **The same analysis as shown in panel a but for DUSs that are present in at least one strain of *Neisseria meningitidis *but are absent in both *N. gonorrhoeae *and *Neisseria lactamica*. Most of these DUSs are not expected to be ancestral. The degeneracy in this case was measured in the *N. lactamica *sequences facing the *N. meningitidis *DUS, and is similar when *N. gonorrhoeae *is used instead. **(c) **Weblogo [87] of the degeneracy of DUS sites in one *N. meningitidis *strain. A weblogo is a graphical representation of a multiple sequence alignment in which the height of the bases in each position indicates their relative frequency, whereas the overall weight of the stack indicates the conservation of that position in the motif. Similar weblogos are found for the other genomes.

We then analyzed the columns in the alignment corresponding to DUSs present in some genomes but not in all. We first considered DUSs present in all except one genome. We found many elements that contained a single mismatch with the DUS consensus sequence (termed DUS-1), and very few cases of complete deletion of the DUS (see Figure 4a for *N. gonorrhoeae *data). This finding might be compatible with the proposed theory that DUSs arose by point mutation, but it is also compatible with simple mutation selection balance, in which mutations resulting in DUS degeneracy are slightly deleterious and thus constitute rare polymorphisms. The second most frequent nucleotide at each position of the 10-nucleotide DUS was then identified as the nucleotide exhibiting the greatest transition frequency in the genome [38] (Figure 4c). Naturally, this is also in agreement with both hypotheses, namely DUS arising by point mutation and being in mutation selection balance.

It might be argued that the frequency of DUS-1 elements suggests that if DUSs are positively selected, then selection is very weak. However, it must be noted that there are 30 different DUS-1 sequences and only one DUS sequence. Under mutation selection balance, the frequency of DUS/DUS-1 is given by $me^{2N_{e}s}$[43], where m is thus 1/30, N_e _the effective population size, and s the selection coefficient (the fractional advantage of DUS over DUS-1). The effective population size was estimated at 10^5 ^in *N. meningitidis *using the population mutation rate (2N_e_μ) of 3 * 10^-2^, as consistently found by Jolley and coworkers [42], and the average wild-type mutation rate (μ) of about 1.5 * 10^-7^, as found by Bucci and colleagues [44]. The observed ratio DUS/DUS-1 of about 2.4 in the aligned regions leads to a coefficient of selection of about 2 * 10^-5^. Usually, one considers that a mutation will tend to escape drift if 2N_e_s is greater than 1. In this case, one obtains an 2N_e_s of about 4, which is sufficiently large for purifying selection to be effective and for DUSs to be under mutation selection balance [43].

### DUSs arise by recombination

**Figure 5:**
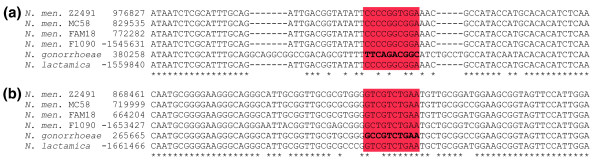
DUS alignment sites. Two examples of aligned DNA uptake sequence (DUS) sites in the multiple alignment of the genomes. The red rectangle surrounds the columns that delimit the DUS sequence, which is presented in bold. The positions are positive if the sequence is in the published strand and in negative if they are in the complementary strand. **(a) **A region of the multiple alignment containing the 3'-TTCAGACGGC-5' reverse complement of the DUS exclusively in *Neisseria gonorrhoeae *FA1090. **(b) **A second region from the multiple alignment containing the 3'-GCCGTCTGAA-5' DUS sequence in *N. gonorrhoeae *FA1090, and showing an altered DUS with one substitution in the remaining sequences.

We then investigated the role that recombination plays in replacing mismatched DUSs with perfect ones and in creating new DUSs in a particular location of the genome *de novo*. First, we took all positions from the multiple alignment (namely, from the core genome), where we did not find a DUS in *N. gonorrhoeae *or in *N. lactamica*, but we did find one in at least one strain of *N. meningitidis*. We then computed the number of mismatches of the *N. gonorrhoeae *and *N. lactamica *sequences aligned with this DUS. If DUSs were created only by point mutations, then we should observe only very few elements with multiple mismatches, because at this low level of divergence very few point mutations are expected to accumulate in 10-nucleotide loci (Figure 4b). Alternatively, DUSs may be created by recombination and stabilized by mutation selection balance. In this case, the majority of DUS-1 elements reflect mutation selection balance, in which selection favors DUSs but random mutations will constantly create DUS-1 elements. The latter have low probability of fixation but may remain in populations for some time as slightly deleterious polymorphisms. If this scenario is correct, then one would expect to find some DUSs matching DUS-1 elements (reflecting the balance), whereas other DUSs should match sequences many mismatches away (reflecting origin by recombination) and few DUSs matching sequences with intermediary divergence. Indeed, the majority (>60%) of these DUS aligned with either a DUS-1 or with totally unrelated sequences (for example, see Figure 5), frequently matching a series of gaps in the other genomes. The large number of *N. meningitidis *DUS facing sequences with no similarity to DUSs in the genomes of the other species (Figure 4b) is demonstrative of frequent DUS acquisition by recombination in genomes, rather than exclusively (or at all) by point mutation.

If DUSs are under mutation selection balance, and mutation rates are the same in every genome, then genomes with more DUSs result from stronger purifying selection on DUS (counter-selection of cells containing degenerated DUSs). If DUSs are under molecular drive, then more frequent conversion would lead to more DUSs. In both cases, DUSs should be more conserved in such a genome (there should be fewer DUS-1 elements for every DUS). This is because, as mentioned above, DUS-1 elements have been found not to increase transformation rates relative to no DUSs at all [11]. The *N. lactamica *genome contained the highest number of DUSs in the aligned regions and yet the lowest number of DUS-1 elements (Table 1). We detected 308 DUSs in the conversion fragments identified in *N. lactamica*. This is significantly more than the 270 that were expected, given the size of these regions and the DUS density in the multiple alignment (*P *< 0.01, χ^2 ^test). Thus, in the genome with the higher density of DUSs, these motifs are more conserved and we find the smallest gene conversion fragments along with an over-representation of DUSs in the fragments. Collectively, this evidence points toward DUS integration in the genome by recombination and subsequent selection to allow conspecific natural transformation.

### DUSs do not promote genetic diversity in *Neisseria*

Because it is a widely held belief that the evolutionary role of natural transformation is to generate diversity in genomes, we were surprised to discover that predicted horizontally transferred regions contain very few DUSs. Laterally acquired genes of *N. meningitidis *Z2491 and MC58 were collected from the HGT-DB database [45] and scanned for the presence of DUSs. These predicted recently transferred genes with low %G+C amounted to 5.6% of the Z2491 genome and 7.1% of the MC58 genome, but they contained only 2% (38) and 2.6% (51) of the total number of DUSs, respectively (*P *< 0.001, χ^2 ^test). These horizontally transferred regions thus held significantly fewer DUSs as compared with the genome average, suggesting that DUS-mediated transformation is not associated with gene flux, which may arise by other means such as transduction.

The previous analysis was conducted in genes with peculiar sequence composition, which typically represent horizontally transferred genes from distant species. However, because these sequences are often A+T rich [46], they might be expected to lack the G+C-rich DUS sequences. Furthermore, methods based on atypical sequence composition miss the transfers from genomes of similar oligonucleotide composition. Therefore, we conducted a more rigorous and conservative analysis of transfer by using the presence and absence of sequences within the six genome sequences. We first counted the number of DUSs in the regions not aligned by M-GCAT (in the regions containing genes that are not ubiquitous in the clade). Because these genomes diverged recently and exhibit few substitutions (average 85% identity in DNA sequences), point mutations cannot account for the divergence of unaligned regions. These can thus be assumed to contain the horizontally transferred genes and the genes that were lost in some, but not all, genomes. Only about 10% of the total DUSs were located there, although they account for 17.5% of the sequence (*P *< 0.001, binomial test).

The results described above suggest that selection for genetic novelty is not associated with the selection for DUSs because recent acquisitions under-represent these motifs. We thus conducted a strict test to check whether DUSs are under-represented in both new laterally transferred sequences and in the sequences that, although present in the ancestral genome, were recently lost in an extant one. The *N. meningitidis *Z2491 genome was used as a reference and the 34 genes with more than 100 codons that were absent in all other genomes were analyzed (the length threshold was set to avoid the uncertainties associated with the annotation of small genes). Most of these recently acquired genes have no known function, and they are all devoid of DUS elements. The probability of finding no DUSs in such a large set of genes by stochastic effects is very small (*P *< 0.001, χ^2 ^test), both when controlling for the number and for the length of these genes. In comparison, the ubiquitous genes contain an average of 0.4 DUSs per gene. We then identified the genes that were present in the *N. meningitidis *Z2491 and all other genomes except in the *N. meningitidis *MC58 genome. The 29 genes encountered were present in the ancestral genome and recently lost in *N. meningitidis *MC58. These genes were also totally devoid of DUSs, which is significantly different from the expected number (*P *< 0.001, χ^2 ^test). We found the same results when performing the same analysis using *N. meningitidis *MC58 genome as a reference (data not shown). DUSs were completely absent from very recently gained genes, suggesting that DUSs are of minor importance in gene acquisition. Lost genes also lacked DUSs, indicating that DUS absence may render a sequence more prone to be lost than DUS-containing sequences.

Many genes in *N. meningitidis *are highly variable because they are under selection for diversification. These genes may belong to the core genome, but their expression or functional pattern changes rapidly because of replication slippage within short repeats contained in their coding or regulatory sequences or because of homologous recombination with homologous (pseudo) genes. Although it may not be simple to identify these genes, they usually share one or both of the following characteristics: they correspond to contingency loci [31], or they correspond to outer membrane or extracellular proteins [47]. We therefore complemented a list of contingency loci in *N. meningitidis *[30] with genes having identifiable peptide signals using SignalP [48], which resulted in a subset containing between 56 and 92 genes, depending on the genome. We then identified DUSs in these genes and checked whether they contained more or fewer DUSs than the average gene in the genome when controlling for gene length. We consistently found lower densities of DUSs in these genes than in the average gene (Table 2). This is in agreement with our prior analyses, suggesting that there is no evidence for the association of selection for diversification with selection for recombination by natural transformation.

**Table 2:** Distribution of 10-mers DUS in the genes showing phase variation and/or containing signal peptides

	Number of genes	Number of DUSs	Expected	*P *value
*N. meningitis *Z2491	74	13	25	0.01
*N. meningitis *MC58	61	6	22	0.0004
*N. meningitis *FAM18	72	20	27	0.16
*N. meningitis *8013	53	3	16	0.0008

Expected values were computed taking into consideration the number and size of these genes relative to the average gene in the genome. *P *values were computed using χ^2^. DUS, DNA uptake sequence.

### DUSs and USSs are located in permissive regions of their core genomes

**Figure 6:**
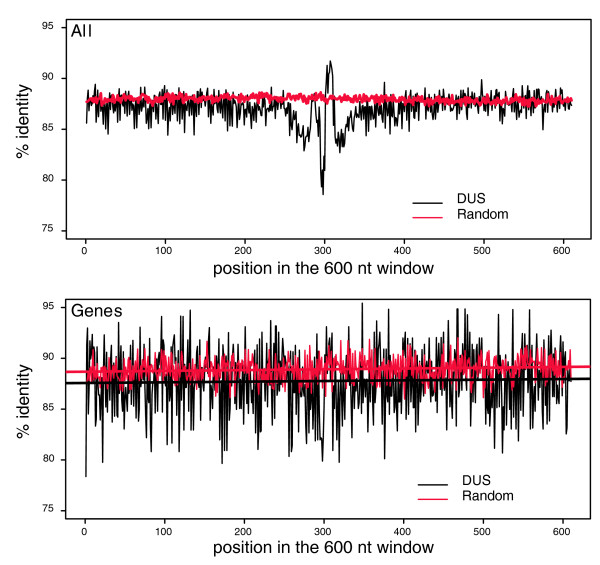
Within the core genome the DUS proximal regions accumulate more substitutions. The black line represents the percent identity for regions surrounding all exactly conserved DNA uptake sequence (DUS) in the multiple alignments. The red line corresponds to the percentage identity for regions surrounding randomly selected DUS-less sites in the multiple alignment. **(a) **All DUSs analyzed. **(b) **Only DUSs contained inside protein coding sequences.

DUSs were over-represented in the 79 co-linear regions common to all of the neisserial genomes. To analyze the exact distribution of DUSs in the core genome, all DUSs along the M-GCAT aligned regions were identified (Table 1). The analysis was then focused on DUSs by sampling 300-nucleotide regions upstream and downstream of all DUSs in the M-GCAT alignment. As a control, we conducted exactly the same analysis for regions randomly selected from co-linear regions from the multiple alignment that did not contain DUSs, in a similar size sequence window. A preliminary analysis indicated that DUS-negative regions were less conserved than DUS-containing regions, but more careful examination showed that this was due to an undue effect of some large indels. Indels appear in this analysis as highly divergent regions, but the reasons for this are probably associated with the above-mentioned lack of DUSs in gained and lost regions. We therefore eliminated from our subsequent analysis the gapped columns (Additional data file 3). As a result, the sequence identity values reported were calculated only using substitutions. The remaining alignments are expected to be a more accurate representation of the core genome. On average, DUS-containing regions exhibited 15% divergence (85% sequence identity; Figure 6). The DUS-negative regions exhibited 13% divergence. Thus, DUS-containing regions are about 15% more divergent. Because the majority of DUSs are located in intergenic regions, and because these regions evolve faster, this result might be expected even if there were no DUSs. To control for the effect of high substitution rates in intergenic regions, we assessed DUSs inside genes only and compared them with random regions that were also centered inside genes. Again, the DUS-proximal regions in genes were found to be around 15% more divergent than DUS-negative regions in genes (exhibited 87% and 89% sequence identity, respectively). This shows that DUSs are located in slightly more permissive regions within the conserved core genome, even though DUSs themselves are highly conserved (Figure 6).

For a preliminary comparison, we conducted a similar analysis on the alignment of the four genomes of *H. influenzae *(Additional data files 6 and 7). The results were indeed similar, showing that the USS-flanking regions were less conserved than USS-negative regions in genomes, and that USSs themselves - like DUSs - are more conserved. Thus, DUSs and USSs are associated with regions conserved in all genomes and, within these regions, they are located in the parts that were permissive to substitutions.

### Modeling the effect of DUS proximity

**Figure 7:**
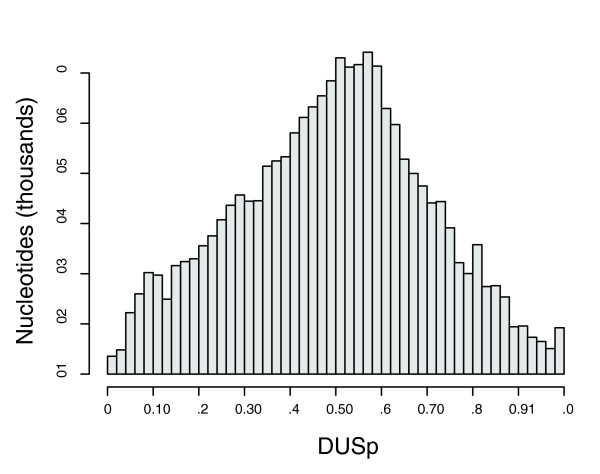
Distribution of the values of DUSp among all positions in the *N. meningitidis *Z2491 genome. All DNA uptake sequence (DUS) positions in *N. meningitidis *Z2491 were excluded from the histogram. DUSp, DUS proximity.

The previous analyses of core genes and diversifying genes accounted for the presence of DUSs inside genes. However, a DUS that is not intragenic but is contiguous to a gene may still significantly affect the potential for uptake and recombination of that gene. This highlights the relevance of studying complete aligned genomic data, relative to simple alignments of orthologous genes. In order to analyze the effect of DUS proximity on genes and conversion events, we have created a measure termed DUS proximity (DUSp). DUSp accounts for the distance of a nucleotide to the average of the closest upstream and downstream DUSs, taking into account the distribution of sizes of the conversion fragments for each genome. DUSp measures the potential effect of the closest DUS on a neighboring nucleotide by facilitating DNA uptake and thus recombination with exogenous DNA. If a position is very close to a DUS, then the effect of DUSs on recombination at that position will be high since most conversion fragments that are taken in because of this DUS will be sufficiently large to lead to recombination at that position. If a position is at a distance from the closest DUS that is larger than the size of the largest conversion fragment found with GENECONV, then recombination at this position will not benefit from the presence of DUSs. In short, a nucleotide contiguous to a DUS has maximal DUSp, and one very distant has a DUSp close to zero (see Materials and methods, below, for details). We exemplify the values taken by this measure with the histogram of DUSp in the genome of *N. meningitidis *Z2491 (Figure 7). This shows that very few positions in the genome are sufficiently far away from DUSs to be almost unaffected by their presence; for example, fewer than 1% of positions have a DUSp lower than 0.05.

We then checked whether positions in genes putatively under selection for diversification had an average DUSp lower than the rest. In *N. meningitidis *Z2491, DUSp in these genes averaged 0.39 versus 0.49 in the remaining genes (*P *< 0.001, *t*-test). In *N. lactamica *the results were similar, with average DUSp values of 0.26 and 0.30, respectively (*P *< 0.001, *t*-test). The DUSp values are lower overall in *N. lactamica *than in the other genomes because of the smaller conversion fragments and in spite of higher DUS density. The use of our DUSp index confirms that selection for diversification in rapidly evolving genes related to fitness and virulence is not associated with selection for recombination by natural transformation.

To quantify the association between the distribution of DUSs and sequence conservation, we also computed the correlation between DUSp and sequence identity. This analysis is somewhat delicate because most columns in the multiple alignment are strictly identical between all genomes, whereas the remaining ones are bi-allelic (Additional data file 4). Thus, we focused the analysis on calculating the average DUSp for all nucleotides in *N. meningitidis *Z2491 (Figure 7) and classified all aligned positions in *N. meningitidis *Z2491 as changed if they differed from the consensus, or as not changed if they agreed with the consensus. The average position in this genome had a DUSp of 0.51 for changed sites and 0.49 for the others. The difference is small, but it means that across the aligned *N. meningitidis *Z2491 genome changed sites are on average 40 nucleotides closer to DUSs than the others, and the difference is highly significant (*P *< 0.001, analysis of variance). Thus, DUSs are associated with permissive regions that exhibit higher sequence diversity, even though DUS themselves are highly conserved.

## Discussion

The use of multiple genome alignments facilitated elucidation of the role played by DUSs in genome evolution in greater detail than was possible in previous studies. We have thus been able to show that DUSs are associated with recombination hotspots (with regions of increased recombination rates). First, their spacing matches the length of conversion fragments. Second, the analysis of recently acquired DUSs identifies many cases in which the motif region matches homologous regions lacking any motif resembling a DUS, which suggests insertion by recombination and not by point mutation. Third, *N. lactamica *has smaller conversion fragments and more tightly spaced DUSs, and these conversion fragments over-represent DUSs. This association between recombination and DUS distribution may be caused by selection for recombination, by selfish molecular drive, or by both.

Simple preliminary analysis of the degeneracy of DUSs allows determination of coefficients of selection compatible with DUSs being under mutation selection balance. An interesting case is provided by the analysis of *N. lactamica*, which has a higher DUS/DUS-1 ratio, suggesting that stronger selection for DUSs results from smaller conversion fragments. This is in accordance with the experimental observations that natural competence in *N. lactamica *is more specific (as opposed to being genus specific) than competence in *N. meningitidis *and *N. gonorrhoeae*, which exhibit only DUS dependency irrespective of the source of DNA [49]. It is thus tempting to speculate that more discriminating transformation and smaller conversion segments cause selection or molecular drive for a higher density of DUSs.

Because DUSs are markers of recombination resulting from natural transformation, their role must be understood in the light of the multiple theories for the evolution of transformation: sex for the acquisition of heterologous sequences (horizontal transfer *sensu strictu*); sex to allow diversification of quickly evolving functions (for example, virulence factors); sex as a source of food; sex as a source of template for DNA repair; or sex as a mechanism allowing allelic (homologous) recombination to purge deleterious mutations and avoid clonal interference [50-54]. Some of these hypotheses are difficult to distinguish from the point of view of DUS distribution and evolution. However, one can easily distinguish the first two from the remaining ones because they lead to very different expected distributions of DUS elements.

Horizontally transferred regions *sensu strictu *have very few DUS elements, and recent insertions have no DUSs at all, suggesting that these sequences had no DUSs at the time of transfer. In addition, we find that conversion fragments are small, which should severely limit the extent of co-transfer of heterologous sequences with DUSs. Therefore, our data are in clear disagreement with the idea that the primary role of transformation is to mediate the horizontal transfer of new genetic information. Although it had been thought that transformation is the major vehicle of lateral transfer in *Neisseria *[55], recent data show that extensive genetic variation originates from phages and other mobile elements [56,57]. In fact, most well documented incidences of HGT *in Neisseria *are the result of illegitimate not homologous recombination [51,57,58]. This does not mean that transformation never leads to HGT (for example, some recently horizontally transferred elements flanked by DUSs have raised speculations that they arose by natural transformation) [59,60].

It has often been suggested that transformation allowed quick diversification of genetic information involved in virulence. However, here we find that even the core genes known to be under selection for diversification contain fewer DUSs than expected. This further argues against a role of DUSs and natural transformation in selection for genetic diversification, either through horizontal transfer of new functions or through variation in extant genes under selection for diversification.

If the purpose of bacterial sex were to feed on DNA, then DUS specificity would be clearly deleterious, because it prevents most DNA from entering the cell. Even if a DUS were present by chance, this would occur more frequently in G+C-rich genomes (because DUSs are G+C rich), whereas the most required nucleotide in cells is A because of the energetic metabolism [61]. As a result, and because degeneracy in protein-DNA interactions is the norm, bacteria exhibiting lower DUS specificity should arise and quickly out-compete DUS-specific *Neisseria*. It follows either that DUS specificity is highly deleterious, and one might wonder why degeneracy has not evolved, or that nutrient acquisition is not the main purpose of sex in *Neisseria*. In light of the observed association between DUSs and recombination, the second hypothesis seems more plausible.

We show that DUS regions have the same average evolutionary history as the core genome. Nevertheless, DUSs are highly conserved despite being located in regions slightly more divergent than the average core genome. This data and the link between DUSs and conversion fragments is thus consistent with the scenarios of sex for repair, for allelic re-assortment, or for DUS being selfish sequences under pure molecular drive. Because recombination is mutagenic [62], one would expect regions close to DUSs to be evolving quicker than the average core genome, as observed. Because many DUSs arise by recombination, one would also expect their concentration to be higher in more plastic regions of the core genome, as observed. Thus, in all three evolutionary scenarios one would expect DUSs to be highly conserved, but more frequent in the permissive regions of the core genome, while rare in the accessory genome.

Recent findings show that competence for natural transformation in *Bacillus subtilis *stops growth [63] and is associated with the expression of proteins involved in recombination and repair, and that these proteins co-localize with transformation proteins at the cellular poles [64,65]. This suggests a strong link between transformation, recombination, and repair. We previously showed that genome maintenance genes are enriched in both DUS in *Neisseria *and USS in the phylogenetically distant Pasteurellaceae, suggesting that transformation mediates DNA repair and conservation rather than diversification of lineages [10]. The over-representation of DUSs and USSs in genome maintenance genes may reflect selection for facilitated recovery of genome preserving functions and co-evolution between these processes and specific transformation. Although DNA uptake increases upon UV mutagenesis in *B. subtilis *[50], competence for transformation is not found to be regulated by DNA damage in *B. subtilis*, in *H. influenzae *[66,67], or in the constitutively competent *Neisseria *spp. In fact, *N. gonorrhoeae*, and quite possibly other *Neisseria *spp., is polyploid [68] and may use another copy of the chromosome to repair DNA damage by homologous recombination. It is therefore unclear to what extent transformation alone plays a role in the repair of DNA lesions.

Transformation allows the re-assortment of alleles in populations. In this sense, transformation can both reduce clonal interference, the competition between adaptive mutations in different clones, and efficiently purge deleterious mutations [69]. We find that DUSs are missing in both ancient genes of the neisserial clade that were recently lost in one genome and in large gaps in the multiple alignments. This is in accordance with transformation allowing the recovery of inactivated or completely lost genes [10,70]. A DUS might not change the probability of deletion of a gene, but once lost the presence of DUSs in a gene increases the probability that it will be restored by natural transformation. Sex by transformation in *Neisseria *could thus have evolved to deal with the vast amount of deleterious polymorphisms that result from the mechanisms aiming at rapid sequence diversification, for example for the evolution of virulence, such as intrachromosomal recombination and mutator periods. Repeats in *Neisseria *account for approximately 20% of the genome [29], and some of them, such as Correia elements [71] and insertion sequences [28], are highly dynamic. Interestingly, other pathogens that also generate variability through frequent intrachromosomal (homologous or illegitimate) recombination and/or high mutation rates, such as *H. influenzae *[72,73] and *Helicobacter pylori *[74], are also naturally competent bacteria. Furthermore, several transformable bacteria that do not have sequence-specific transformation systems, such as *B. subtilis*, *Streptococcus pneumoniae*, and *H. pylori*, have other genetic or ecological mechanisms ensuring that natural transformation is induced when the likelihood for uptake of conspecific DNA is very high [75-78]. Indeed, our previous and other analyses [10,16] suggested that patterns of DUS and USS evolution are similar in *Neisseria *and *Haemophilus*. In both cases one expects that variability generated by intrachromosomal recombination will lead to deleterious mutations that would quickly accumulate in lineages in the absence of recombination, a phenomenon known as Muller's ratchet. This would fit well with recombination having a role in the maintenance of the core genome.

In order to elucidate the role of DUS-specific transformation, it is vital to determine exactly how DUSs are created *ex nihilo*, which is the mechanism of DUS specificity, and how intense is the influence of molecular drive. We showed that DUSs arise frequently in genomes and that the presence of DUSs is associated with gene conversion events. However, originally these DUSs must have been created by some mechanism. Because the exact site of many new DUSs has sequences of very weak similarity in the other genomes, point mutation is an unlikely candidate for the origin of new DUSs in these populations. *Neisseria *both take up and export DNA; therefore, harboring DUSs in a genome increases the probability for DNA propagation (it increases the fitness of the genes in its neighborhood). In this case selfish molecular drive would most frequently be in accordance with the interest of the cell, which is to filter out nonhomologous DNA and to select for uptake sequences associated with the core genome. In this sense, an understanding of how DUS specificity works will allow an appreciation of its possible evolutionary flexibility as well as its evolutionary history. Did the original system require a perfect DUS sequence, which would severely restrict the range of natural transformation? Alternatively, did DUS specificity co-evolve with the increase in the density of DUSs in genomes? In that case, did the positive feedback of molecular drive allowed faster evolution toward DUS stringent specificity? Both scenarios are plausible on theoretical grounds [79] but have radically different consequences for the theories aiming to explain the role of natural transformation. Our data suggest that the role of DUS-mediated natural transformation in virulence may not be the most commonly invoked. Instead of providing novelty in genetic repertoires, natural transformation may be a mechanism to tackle the side effects of the vast generation of genetic hypervariability that constantly takes place in neisserial genomes. Therefore, an understanding of the evolutionary role of DUS-specific natural transformation may highlight how the bacteria face both the needs for variability in its interaction with hosts and the commitment to preserve the core genome.

## Materials and methods

### Genes and genome sequences

We analyzed the genome sequences of six *Neisseria *spp. and four *Haemophilus influenzae *strains. Four of the neisserial and all of the Pasteurellaceae genome sequences and their annotations were obtained from GenBank Genomes: *N. meningitidis *Z2491, serogroup A (NC_003116) [27]; *N. meningitidis *MC58, serogroup B (NC_03112) [28]; *N. meningitidis *FAM18 serogroup C (NC_03221) [30]; *N. gonorrhoeae *FA1090 (NC_002946, unpublished); *H. influenzae *Rd KW20 (L42023.1) [80];*H. influenzae *strain 86-028NP (CP000057.1) [81]; *H. influenzae *PittEE (CP000671.1) [82]; and *H. influenzae *PittGG (CP000672.1) [82]. The sequence data for *N. lactamica *ST-640 were produced by the Pathogen Sequencing Unit at the Sanger Institute (Cambridge, UK). The sequence and annotation data for *N. meningitidis *8013 serogroup C were provided by the unit 'Génomique des microorganismes pathogènes' from Institut Pasteur (Paris, France).

### Definition of sets of orthologous genes

A preliminary set of orthologs was defined by identifying unique pair-wise reciprocal best hits, with at least 40% similarity in protein sequence and less than 30% difference in length. This list was then refined by combining the information on the distribution of similarity of these putative orthologs and the data on gene order conservation (as in the report by Rocha and coworkers [83]). Because few rearrangements are observed at these short evolutionary distances, genes outside conserved blocks of synteny are likely to be xenologs or paralogs. Hence, we conservatively used the distribution of sequence similarity within reciprocal best hits, together with the classification of these genes as either syntenic or nonsyntenic, to set appropriate lower thresholds of protein sequence similarity between orthologs. We considered two genes to be orthologs if their proteins were at least 85% similar. The definitive list of orthologs for each group was defined as the intersection of pair-wise lists.

### Correlation of predicted HGT regions and DUS content

The six-genome analysis was preceded by mapping of DUS content in predicted HGT regions in the *N. meningitidis *Z2491 and MC58 genomes, as identified in HGT-DB. These are regions with statistical parameters such as G+C content, codon, and amino acid usage deviating from the genome average [45].

### Multiple genome alignment

The M-GCAT genome comparison and alignment tool was used to produce a multiple alignment of the six genome sequences [39]. As output, M-GCAT returns the alignment partitioned into locally co-linear regions or clusters, along with a concatenated version of the multiple alignment that joins all individually aligned co-linear regions. Aligning only locally co-linear regions with strong evidence of homology avoids forcefully aligning potentially nonhomologous sequence. For our comparison, the M-GCAT parameters were configured as follows: q = 100; d = 40000; c = 110; min Anchor length = 0.8 * (Log [S]); min MUM length = 8. The remaining parameters were left as default values. Different values for these parameters will vary the final output. Accordingly, we optimized these values by maximizing the final amount of matches found and percentage of sequence covered by the comparison framework. We used MUSCLE [37] to align the unaligned regions in the M-GCAT comparison framework and to produce the final gapped alignment. After this step, all remaining unaligned regions are lineage specific (they are not in the core genome) and are left unaligned for further inspection. Gblocks [84] was used to calculate the number and identify the regions of conserved blocks in the multiple alignments.

### Phylogenetic analyses

We conducted three separate phylogenetic analyses using three different but partially overlapping datasets: the concatenate of the alignments of the ubiquitous genes; the concatenate of the M-GCAT multiple alignments; and the concatenate of all 1,000 nucleotides regions (± 500 nucleotides on each side) surrounding each DUS present in all of the genomes of the M-GCAT multiple alignment. All analyses were conducted using Tree-Puzzle [85] to generate the matrix of distances by maximum likelihood, with the HKY+Γ model and exact parameter estimates. The trees were then computed using BIONJ [86].

### Gene conversion analysis

To estimate the number and size of gene conversion events between all pairs of these six sequences, we employed the gene conversion detection tool GENECONV [41] on the M-GCAT multiple alignments. The parameters were configured as follows:/mig0.005/g2/dm -Outerseq = off. GENECONV aims to find the most likely candidates for gene conversion events between pairs of sequences in a DNA alignment. It does so by looking for maximal aligned pairs of segments that are unusually similar at a local level. We have also controlled for invariable or highly selected sites by excluding monomorphic sites from the analysis. Candidate events were ranked by multiple comparison corrected *P *values.

### Definition of DUS proximity

The correlation of functional genomic features with DUS presence is complicated by the over-representation of these elements in intergenic regions. Thus, comparing the number of DUSs present in genes neglects the fact that many genes lacking DUSs may have one just after the stop codon. Because we are interested in DUS proximity in relation to DUS-related recombination, we used the distribution of conversion fragment sizes between genomes to compute the probability of a nucleotide being affected by the presence of a neighboring DUS from the point of view of gene conversion. Thus, for a pair of genomes A and B, we compute the cumulative distribution of the sizes of gene conversion fragments given GENECONV (CD). A nucleotide at a distance X from the closest DUS has a score 1 - CD(X), which represents the likelihood that the position will be affected by presence of the closest DUS in terms of engaging into a conversion fragment arising from transformation. Nucleotides far from DUSs will have very low scores, whereas nucleotides close to DUSs will have scores close to 1. We tested some variants of this method, notably by summing the score of the position for the closest downstream and upstream DUSs and by taking the maxima of the two. Both maxima and average approaches give qualitatively comparable results. In the text we focus on the average approach.

### Motif search

All searches for exact and degenerate DUSs in the genome sequences and conserved in the multiple alignment were found using a customized Python script (available upon request). Additionally, the script was used to identify the number of DUSs in coding sequences and calculate levels of DUS degeneracy and percent identity surrounding DUS sites in the alignment.

## Abbreviations

cDUS, composite DUS; DUS, DNA uptake sequence; DUSp, DUS proximity; HGT, horizontal gene transfer; USS, uptake signal sequence.

## Authors' contributions

T Tønjum, OHA and EPCR originated the project. T Treangen, OHA and EPCR conceived, designed, and performed the experiments. All authors analyzed the data and wrote the paper. All authors read and approved the final version of this manuscript.

## Additional data files

The following additional data are available with the online version of this paper. Additional data file 1 provides details regarding genome alignment. Additional data file 2 shows the average nucleotide distance between DUSs. Additional data file 3 details the under-representation of DUSs in strain-specific insertions. Additional data file 4 details the classification of polymorphic sites. Additional data file 5 shows the distribution of the distance between contiguous composite DUSs in the *N. meningitidis *Z2491 genome. Additional data file 6 provides a visual representation of M-GCAT's multiple alignment of four *H. influenzae *genomes. Additional data file 7 shows that within the core genome of *H. influenzae *the USS-proximal regions accumulate more substitutions.

## Supplementary Material

Additional data file 1 Details regarding genome alignment Click here for additional data file

Additional data file 2 The average nucleotide distance between DUSs Click here for additional data file

Additional data file 3 The under-representation of DUSs in strain-specific insertions Click here for additional data file

Additional data file 4 The classification of polymorphic sites Click here for additional data file

Additional data file 5 The distribution of the distance between contiguous composite DUSs in the *N. meningitidis *Z2491 genome Click here for additional data file

Additional data file 6 A visual representation of M-GCAT's multiple alignment of four *H. influenzae *genomes Click here for additional data file

Additional data file 7 This data shows that within the core genome of *H. influenzae *the USS-proximal regions accumulate more substitutions Click here for additional data file
