# Supplementary material for: The impact of the neisserial DNA uptake sequences on genome evolution and stability
Source: Genome Biol. 2008 Mar 26;9(3):R60. doi: 10.1186/gb-2008-9-3-r60 (PMC2397512; doi:10.1186/gb-2008-9-3-r60)
Supplement: Additional data file 1 — Details regarding genome alignment [file gb-2008-9-3-r60-s1.pdf]

Additional Table A1. Multiple alignment details. Details of the multiple alignment of the six *Neisseria* genomes generated with M-GCAT. The global multiple alignment was divided into 79 M-GCAT Clusters (or locally co-linear regions) that are devoid of breakpoints such as rearrangements or large segments of lineage specific sequence. **Alignment Coverage** is the average percentage of each genome covered by the Clusters. **Polymorphic sites** are the number of variable sites in the alignment, and **Mono-morphic sites** represent the number of columns with conserved residues. **Conserved positions** indicates the total amount of columns with conserved residues in the multiple alignment, and **Conserved Blocks** designates the number of blocks of conserved positions occurring in at least four of the six genome sequences (as specified by Gblocks).

**Alignment Length**	2,224,085
**Polymorphic sites**	192,618
**Mono-morphic sites**	1,394,876
**Sites with >0 Gaps**	636,591
**Conserved Blocks**	5,711
**Conserved Positions**	1,571,424
**Alignment Coverage**	82.5%
**Clusters/Co-linear Regions**	79
**Sites per Cluster**	28,153
