# Supplementary material for: The impact of the neisserial DNA uptake sequences on genome evolution and stability
Source: Genome Biol. 2008 Mar 26;9(3):R60. doi: 10.1186/gb-2008-9-3-r60 (PMC2397512; doi:10.1186/gb-2008-9-3-r60)
Supplement: Additional data file 2 — The average nucleotide distance between DUSs [file gb-2008-9-3-r60-s2.pdf]

Additional Table A2- Average nucleotide distance between DUS. ΔDUS are all DUS elements. Δ cDUS is the distance between composite DUS, which correspond to isolated DUS or to 2 inverted copies of a DUS in a transcription terminator. In this last case the distance between the two inverted copies is not counted as the two copies are regarded as one single composite element.

	Δ DUS	Δ cDUS
N. men. Z2491	1154	1469
N. men. MC58	1174	1511
N. men. FAM18	1162	1506
N. men. 8013	1189	1525
N. gonorrhoeae	1096	1372
N. lactamica	995	1280
