# Supplementary material for: The impact of the neisserial DNA uptake sequences on genome evolution and stability
Source: Genome Biol. 2008 Mar 26;9(3):R60. doi: 10.1186/gb-2008-9-3-r60 (PMC2397512; doi:10.1186/gb-2008-9-3-r60)
Supplement: Additional data file 3 — The under-representation of DUSs in strain-specific insertions [file gb-2008-9-3-r60-s3.pdf]

Additional Table A3- Under-representation of DUS in strain specific insertions. We detected a total of 519 strain specific insertions of length >100nt in the M-GCAT alignment. We searched for DUS inside of these insertions and found significantly less DUS than expected (χ^2 ,p < 0.00024). **# of insertions** indicates the # of insertions >100nt specific only to one strain that were found. **Avg. length** is the mean length of all the insertions specific to a given strain, and # **DUS is** the total number of DUS found inside of all of the strain specific insertions in each genome.

	# of strain specific insertions	Avg. length	# DUS
N. men. Z2491	43	434	1
N. men. MC58	40	365	2
N. men. FAM18	63	429	3
N. men. 8013	47	396	4
N. gonorrhoeae	102	370	12
N. lactamica	224	621	94
