# Supplementary material for: The impact of the neisserial DNA uptake sequences on genome evolution and stability
Source: Genome Biol. 2008 Mar 26;9(3):R60. doi: 10.1186/gb-2008-9-3-r60 (PMC2397512; doi:10.1186/gb-2008-9-3-r60)
Supplement: Additional data file 4 — The classification of polymorphic sites [file gb-2008-9-3-r60-s4.pdf]

Additional Table A4- Classification of polymorphic sites. We classified all of the single nucleotide differences for the 192,618 polymorphic sites contained in the multiple alignment**.** Approximately 95% of the polymorphic sites contained only two alleles, and the remaining 5% of polymorphic sites contained three or four alleles. There were a total of 31 different patterns for sites with two alleles, and the 12 most frequent bi-allelic patterns are shown. Pattern labels are as follows: A = *N. meningitidis* Z2491 serogroup A, B= *N. meningitidis* MC58, serogroup B, C= *N. meningitidis* FAM18 serogroup C, D= *N. meningitidis* 8013 serogroup C, G= *N. gonorrhoeae* FA1090, L= *N. lactamica* ST-640.

Rank	Pattern ABCDGL	Inferred Topology	Freq.	%
1	111112	((ABCDG) L)	59810	31.1
2	111121	((ABCDL) G)	30698	15.9
3	111122	((ABCD)(GL))	16397	8.5
4	121111	((ACDGL) B)	8012	4.2
5	211111	((BCDGL) A)	7561	3.9
6	112111	((ABDGL) C)	6756	3.5
7	111211	((ABCGL) D)	6317	3.3
8	121122	((ACD)(BGL))	3025	1.6
9	121112	((ACDG)(BL))	2951	1.5
10	112211	((ABGL)(CD))	2719	1.4
11	211112	((BCDG)(AL))	2674	1.4
12	111212	((ABCG)(DL))	2575	1.3
