# Supplementary material for: The impact of the neisserial DNA uptake sequences on genome evolution and stability
Source: Genome Biol. 2008 Mar 26;9(3):R60. doi: 10.1186/gb-2008-9-3-r60 (PMC2397512; doi:10.1186/gb-2008-9-3-r60)
Supplement: Additional data file 5 — The distribution of the distance between contiguous composite DUSs in the N. meningitidis Z2491 genome [file gb-2008-9-3-r60-s5.pdf]

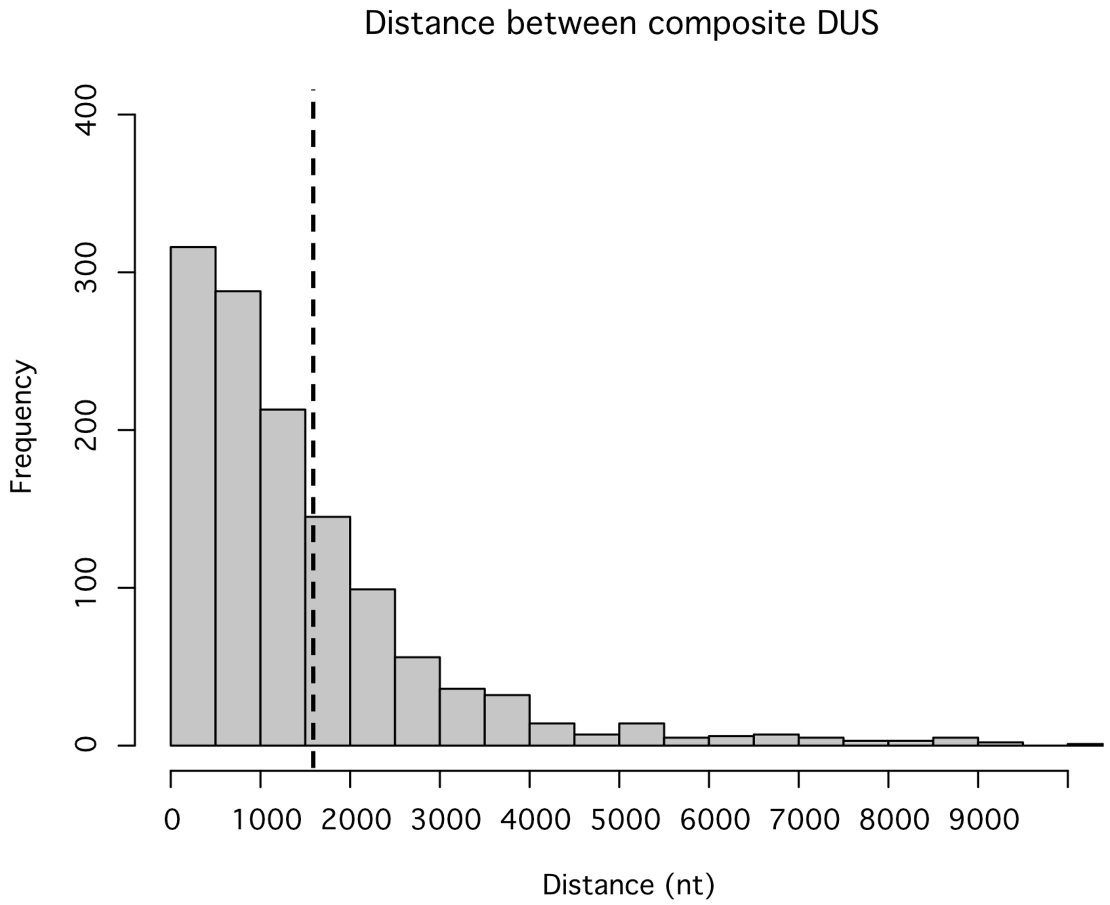

Additional Figure A1 – Distribution of the distance between contiguous composite DUS in the *Neisseria meningitidis* Z2491 genome.
