# Supplementary material for: The impact of the neisserial DNA uptake sequences on genome evolution and stability
Source: Genome Biol. 2008 Mar 26;9(3):R60. doi: 10.1186/gb-2008-9-3-r60 (PMC2397512; doi:10.1186/gb-2008-9-3-r60)
Supplement: Additional data file 6 — A visual representation of M-GCAT's multiple alignment of four H. influenzae genomes [file gb-2008-9-3-r60-s6.pdf]

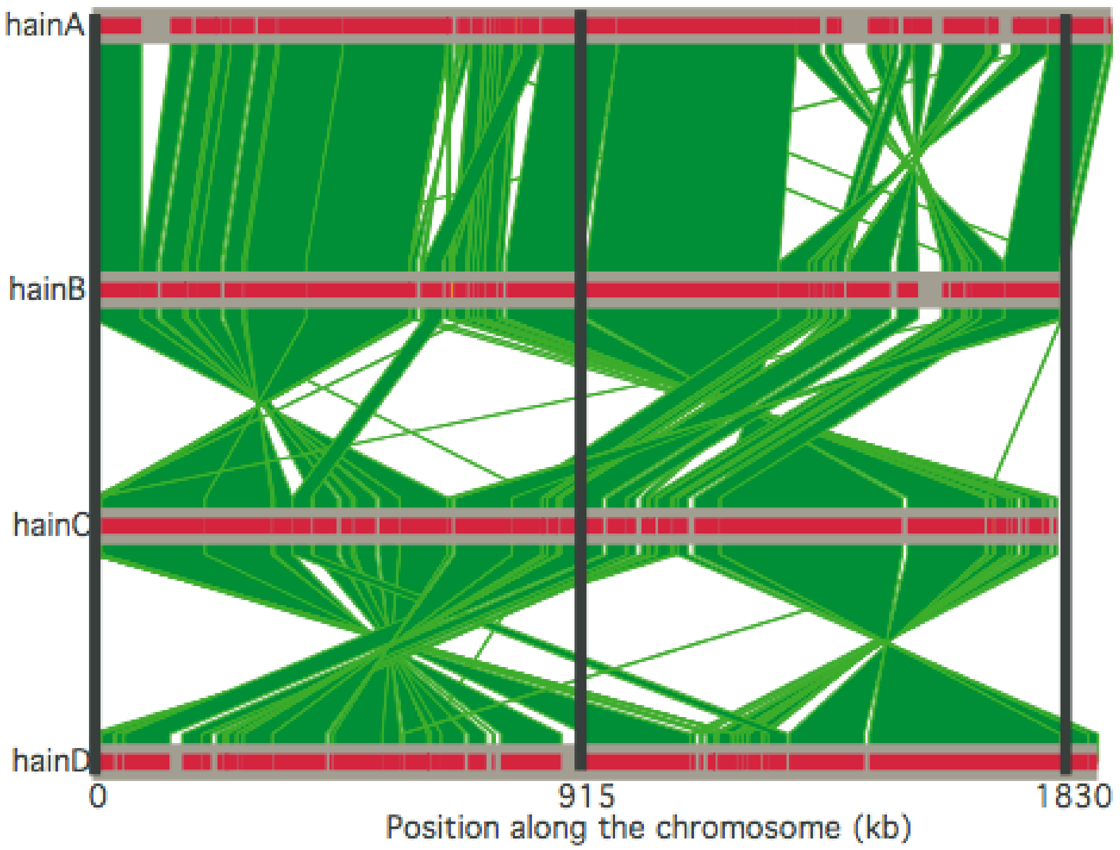

Additional Figure A2 – Visual representation of M-GCAT's multiple alignment of four *Haemophilus influenzae* genomes. **hainA**: *H. influenzae* strain 86-028NP (CP000057.1); **hainB**: *H. influenzae* Rd KW20 (L42023.1); **hainC**: *H. influenzae* PittEE (CP000671.1); **hainD**: *H. influenzae* PittGG (CP000672.1).There were 53 M-GCAT Clusters of average length 32,060 nt. In total, 91.3% of the original genome sequences are covered by the multiple alignment, and 8.7% was left unaligned.
