# Supplementary material for: The impact of the neisserial DNA uptake sequences on genome evolution and stability
Source: Genome Biol. 2008 Mar 26;9(3):R60. doi: 10.1186/gb-2008-9-3-r60 (PMC2397512; doi:10.1186/gb-2008-9-3-r60)
Supplement: Additional data file 7 — This data shows that within the core genome of H. influenzae the USS-proximal regions accumulate more substitutions [file gb-2008-9-3-r60-s7.pdf]

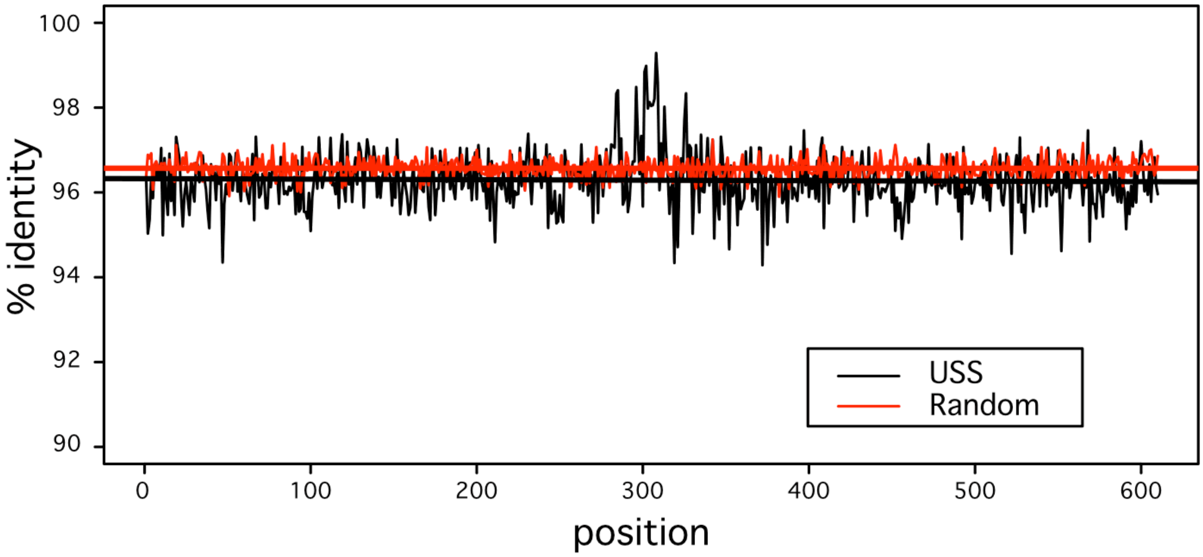

Additional Figure A3 - Within the core genome of *Haemophilus influenzae* the USS-proximal regions accumulate more substitutions. The black line represents the percent identity for regions surrounding all exactly conserved DUS in the multiple alignment. The red line corresponds to the percent identity for regions surrounding randomly selected DUS-less sites in the multiple alignment.
